# Supplementary figures and images for: HaMADS3, HaMADS7, and HaMADS8 are involved in petal prolongation and floret symmetry establishment in sunflower (Helianthus annuus L.)
Source: PeerJ. 2024 Jul 2;12:e17586. doi: 10.7717/peerj.17586 (PMC11225715; doi:10.7717/peerj.17586)

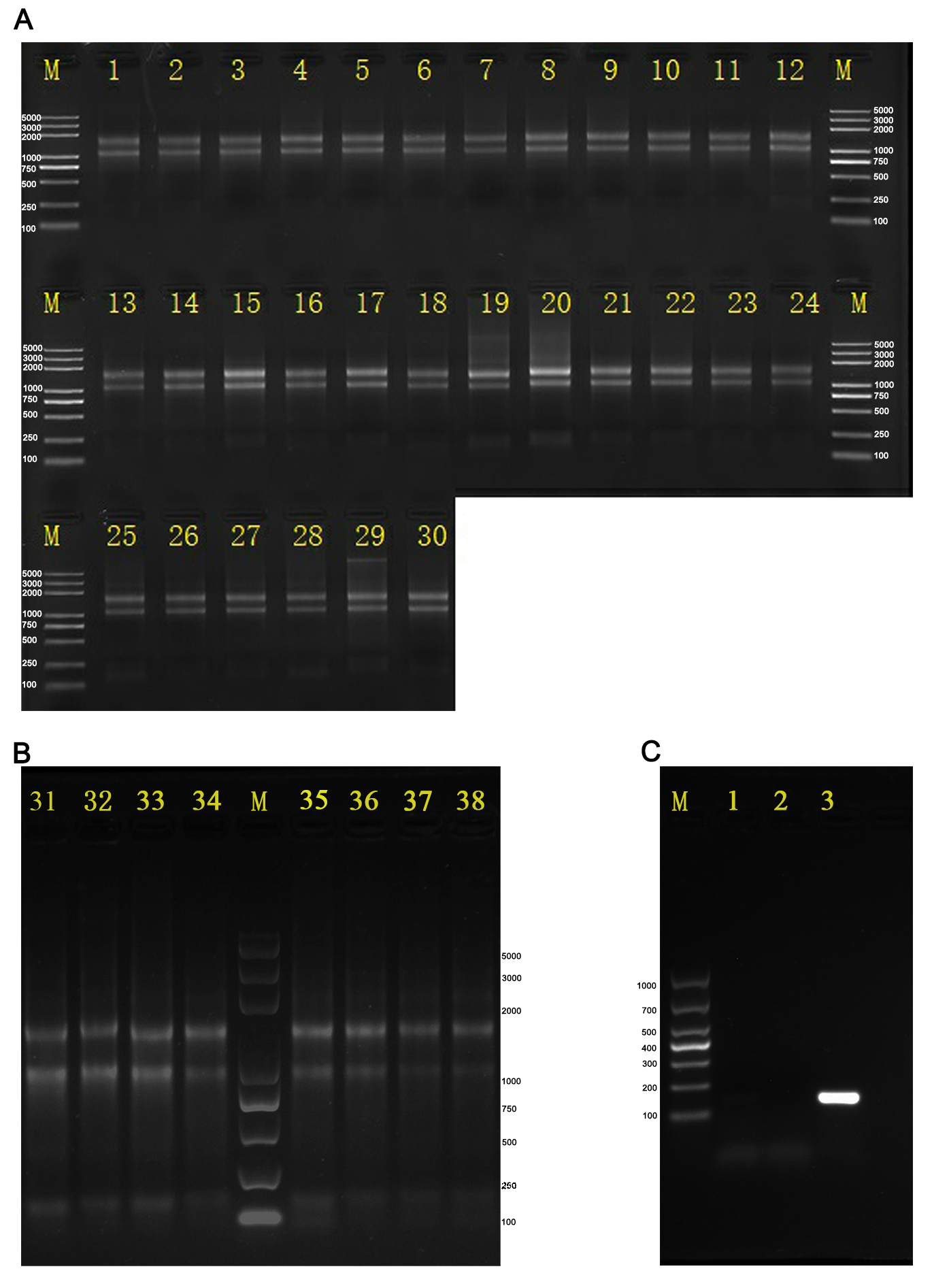

Supplement: Supplemental Information 1 — (A) and (B) Electrophoresis analysis of RNA. M: 5 kb marker. 1: root. 2: stem. 3: leaf. 4: flower. 5: disc floret of WT at 25 DPB. 6: disc floret of lpm at 25 DPB. 7: disc floret of WT at 15 DPB. 8: disc floret of lpm at 15 DPB. 9: disc floret of WT at 5 DPB. 10: disc floret of lpm at 5 DPB. 11: disc floret of WT at 0 DPB. 12: disc floret of lpm at 0 DPB. 13: bract of WT at 5 DPB. 14: bract of lpm at 5 DPB. 15: bract of WT at 0 DPB. 16: bract of lpm at 0 DPB. 17: petal of WT at 5 DPB. 18: petal of lpm at 5 DPB. 19: petal of WT at 0 DPB. 20: petal of lpm at 0 DPB. 21: pistil of WT at 5 DPB. 22: pistil of lpm at 5 DPB. 23: pistil of WT at 0 DPB. 24: pistil of lpm at 0 DPB. 25: petal of WT dics floret at 10 DPB. 26: petal of WT ray floret at 10 DPB. 27: petal of WT dics floret at 5 DPB. 28: petal of WT ray floret at 5 DPB. 29: petal of WT dics floret at 0 DPB. 30: petal of WT ray floret at 0 DPB. 31: Petal in the 1st parastichy of WT floret at 0 DPB. 32: Petal in the 5th parastichy of WT floret at 0 DPB. 33: Petal in the 15th parastichy of WT floret at 0 DPB. 34: Petal in the 19th parastichy of WT floret at 0 DPB. 35: Petal in the 1st parastichy of lpm floret at 0 DPB. 36: Petal in the 5th parastichy of lpm floret at 0 DPB. 37: Petal in the 15th parastichy of lpm floret at 0 DPB. 38: Petal in the 19th parastichy of lpm floret at 0 DPB. (C) Detection of genomic-DNA contamination. M: 1 kb marker. 1: total RNA. 2: RNA treated by gDNA Eraser. 3: cDNA. [file peerj-12-17586-s001.png]

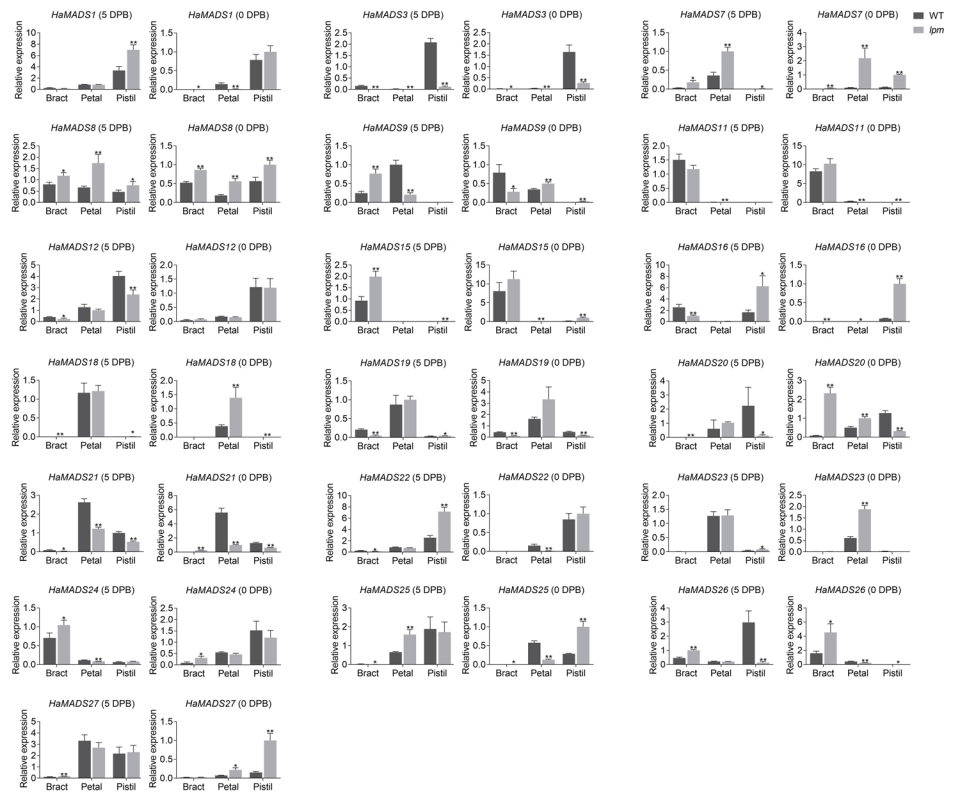

Supplement: Supplemental Information 2 — Note: Each column represents mean ± SEM of 3 technical. “*” represents P < 0.05 and “**” represents P < 0.01 by one-way ANOVA analysis. [file peerj-12-17586-s002.pdf]

**A**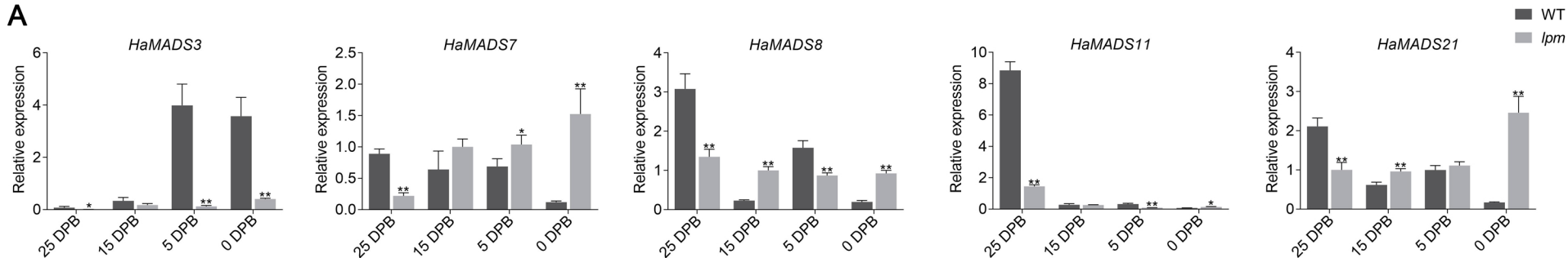**B**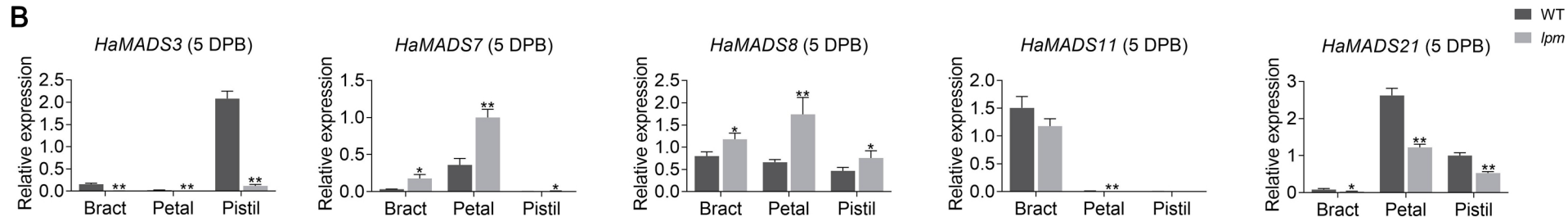**C**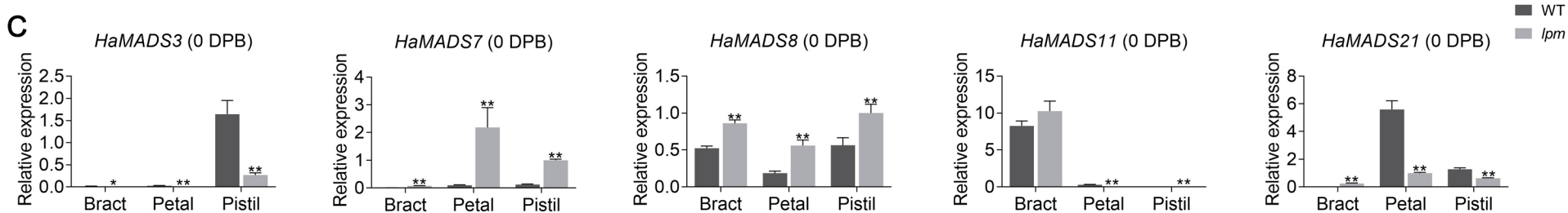

Supplement: Supplemental Information 3 — (A) Expression analysis in florets at different stages. (B) Expression analysis in floral organs at 5 DPB. (C) Expression analysis in floral organs at 0 DPB. Each column represents the mean ± SEM of 3 technical replicates. *P < 0.05 and **P < 0.01 between WT and lpm (one-way ANOVA analysis). [file peerj-12-17586-s003.pdf]
