# Supplementary material for: HaMADS3, HaMADS7, and HaMADS8 are involved in petal prolongation and floret symmetry establishment in sunflower (Helianthus annuus L.)
Source: PeerJ. 2024 Jul 2;12:e17586. doi: 10.7717/peerj.17586 (PMC11225715; doi:10.7717/peerj.17586)
Supplement: Supplemental Information 5 [file peerj-12-17586-s005.docx]

**Supplementary Table 2 The transcripts sequences of 29 MADS-box genes**

>*HaMADS1*

AGCTCATATAGAAGTTCTTAAAGTTAAATGCCCACTAAGGTTTCCATCATGTGCGGTTCTTCTTCGTCGTCATCATCCTCTGGTTGTGGTGGTCAGGCGGGTGGTGgcagcggtggtggtggtggtggtggaggaggagaAGAGAGGAGGAGGAAGACGATGGGTCGGGGGCGGATTGAGATAAAGAAGATTGAGAATGTAAGTAGCCGACAAGTGACATTTTCAAAGCGCCGGGCTGGCCTTATGAAGAAGGCTAAAGAGTTGGCGATTTTGTGTGATGCCGAAGTGGGCGTTATTATTTTCTCGAGCACCGGAAAACTCTATGATTTTGCTAGTTCTCAGTTGCAAAACATTATCTCAAGATACGAAAGGACTTCAAATGGTTGGAATGACCCAATCTCTGGACCACAGcaAGAAGTGTACAACGAGGTAGAAGCTCTGAAGTCTGAAATCATGAAGCTACGGAAGGGACAGAgGTTGATGATGGGAAAAGAACTGGAAGGATTAAACTACAAGGAGCTTGAAAACTTGGAACGCCAACTCCATGATGGCATGTTAGCAGTAAAGAATCGGAAGGATATGGCACTATTGGAAGAAATCGAACAAATAAAACTGAGGGAGCAGAGGACCATGAAAGAAAATGAAGCTTTGAAGAAAGAGATCACCAAGCATCTATATAAATCAACAAGCGGTCATGAAATATGTCTAGTAGGGCGAAAGAGTCCCTTCATAAAGCCGAGCACACTTGCCTATCTTAGTCCGGATAATGGAGATGTCGGTATTTCTTTGCACTTAGGGTTATCACAACGTGATGATCATCAGAAGAGAAAAATTCCAAAAGTAGAGTATGATCCATAAGTTTTGAAGCCAAGTATAATACTTCAAGTTTCAATTCCATGAGTTTTACATTTTGTTATATGTGTGAAGAGATAACTATAATTGCTATATTAAGATCTAAAATCATCTTGGTTGTGTTGTGAGGTTGATTATCATAAGTGTTTATACTTATGACATATAGGGCATCATACACGATTAAATGTCATCGGCTAAAACATTGAAAAGTTAAAGTAACGTTTACCGGGTTTGTTTTGTCA

>*HaMADS2*

CAAGATGGGTGCCATTTCGACTCCACCTTGACCTTACCTCAAAACCTAgttataaactttttttttaaattaacctCTTTAACGAGACAGTGTTTCTGTTGTTTTACGACCCGATGGGGAGAGGGAAGGTTGAACTGAAGCGGATCCAAGACAAAGTGAGCCGCCAAGTCTCCTTCACCAAGCGCCGGAACGGACTGATGAAGAAGGCTCACGAGCTATCCGTGCTGTGCGACGTTGATCTCGCCGTTTTCGTCTTCTCTGGGAGAAACAAGCTCTATGAGTTTTCTACCGGTGACAGCATGCCTGATATCCTTAAGCGCTACGAGGACCACAAGCATGCAGATGAAGCAGTCCGCAGAACTGTACGCGAGGAACTCACGTTACAATATAGGAATGTACGCACCGCTGATGAGTTAACAGAGATGACCCAAAGGCACCTCAATGAGCACAAGGTTCAACAGGCAGATGTCAGCGAGCTCTTTCAGTTAGAGAAACAAATCGAGGCCAATCTCCAACAAGTCAGAACCATAAAAACAAAGTTGATGTTGGGAGTTGTGAAAAACCTGCAAGAGGAGCAAAAACAACTGAGACAAGATAAGCGGTTGATGGAAGGAAAGGTAGCAAATGAGTTGCGGATTTGGGAACGTAACAACTAAACACTGGTTTTGAGGGTGTTGTTATATATCTTTATGATGAAAGCAGGGTGGCTGAATAAAAGGTAATGTTGATGTTTAATGCATCCACTACTTTGTAATGGTTTTCTGTTTTCTGTATCCTTTATTTCAATCGTACTAGTAAGGAAGAAATGTTCTATGTATAATAATGTTGCTATCTCCAAGATATGGTCTTATGTAATATGTCTTTTGGCAATGGAATCTTTGCCTTTTTGTTAGTCA

>*HaMADS3*

ATGGATCTTAGGGTTTTTATTTTAAGACCACTTTACCTTACATTGGAGAGAGAGATTGAACTGAAGTGAGGTGATCGTTTTCTTAAAGGTGTTGGTTTCTTCTTCAAAGGCAAAGTGGAACCATGGGTAGGGGAAAGATTATGATCAAAAGGATCGAAAATGATACGGCTCGACAGGTGACCTTCTGCAAGAGGAGGGGTGGGCTTCTCAAGAAAGCTTGTGAGCTTTCGGTTATGTGCGATGCTGAAATCGCTTTGATTGTCTTCTCCCCACGTGGACGCGTCTATGAGTTTGCCAATAACGACATAAAATCAACTATAGAGAAGTACAAGAGGATAAAATCCGATGAATCAAACACCAATTTCACACCTCAAGAGATTAATGCTCAATATTACCAGGAGGAGGCGAAGAAGCTTCGCCAACAGATAAAAATCCGCCAGAATTCTAACAGGCACCTTAAGGGTGAAGGATTGGATTCTTTGAACATTAACGAGCTGAAGAAACTAGAGACAAAACTTGAGAGAGCAATATCAAGGATCAGATCAAAGAAGCATGACATGATACTTGAAGAAACTGAATCCCTACAGAACAGGGAGCTTGTGCTTGAACACACCAACACAATCCTTCGCTCCAAGATTGCTGAGAATGAGAAGGTGCAGCAGCATTTGGTGGATCAAACTAATGAAGGGTACCGTGTAATAGAAGCATACCTTGCTAGGAGCGCCCTTCAACTTAATATAGGAGGGCCTTTGGAGGATACCCCCACACCAGCACCGGCACCTGCACCTTATTCCCCCTATCCCAACAAGTCTCTTCACATCTGGTGAACAAAGCGGTACTCGAGTAAAGTTTACTTCTATGTAGGACGTTTGGAAGAAGCACTTCGTGAAGTTTGGTTTTGTGTGGTTTTAATTTAAAACGTACCCTAATTAAGTATGAAAACTTAATGTAATGATATGGTCAAGACTATCAACTATGACAACTATATATGATTGTTATTTATGAATTATGGCACTCTAAATAATTTGGGTA

>*HaMADS4*

GGTAAATAACAAAAGAGTACACATTCTTCTGTCTCATCGGTTTCCTTTTATACAACAGTCCGTCTTCAATCTCCCAAGAACCAGACCACTTCCCCCAATTCACCCGTCATCCCTGACATCAACTCAACCAAATAAATTTACTTCAAATCTTGCAAACCCTCCATCGGATCTTCATGGGGTTTTCATCTTCTTTCTGATTTTACAATTTTCTTGAATAATATAACCCACCTTTACctatcatcaccaccaccatctagATCGGATAATAGAGTGAGTTGGGTAAACGAGTGTTCTTGATTTTGTCTAGGGTTTCTAGTTTAAACCACTGATCTATATACCCAATTTTGGTAAAGAAAAATGATGTCCTTTTGTTTCTTTTCTCTTTTGGAAGCTACAAAATTAAATGTTTCTATTCATGGAAAACCCTTGATTTAAACACTGGTTTATTCTATGTTTTCTTgagaaaattattatttttgaaagaaaatttGCCAAAAATAGTGTGTGTGGAACTGGAAAGGATGATGGTTAGGGAAAAAGTACCGATTAAAAAGATTGATAATGCTACAGCAAGAAGGGTGACTTTCTCCAAGAGGAGAAGAGGGTTGTTTAAGAAAGCTGAAGAGCTTTCTGTACTTTGTGATGCTGATGTTGCTGTTATTCTCTTCTCCTCCAATGACAAGCTGTTTCATTACTCCAGTTCAAGTATGGAAGAAGTACTTGAAAGGCGTAGCCTTCACTCAAAAAATCTTGAGAAGTTAAACCAACCTTCTCTTGAGTTACAGCTGGTTGAAGACACCAACTATGCCAACTTAAGCAAAGAAGTTGCAGAAAGAACCCTTCACTTAAGACGGTTGAGAGGGGAGGAGCTGCAGGGTTTAAGTATTGAAGAGTTGCACCAGCTCGAAAAGTCACTTGAAGCTGGATTAAGCCGGGTTGTTGCCAAAAAGGGTGAAGTGATTATGAACGAGATTAATCATCTTCAAGAGAAGGAAGTGAACCTCATGGGGGAGAATGACAAACtaagacaagaaCTTTTGAAGATATCTAATGCTCGGAAGCAAATTATTTGTGATTCTGGTGATGATCGGGAGTCCTCGGTATCTACCGATATCTGCAACTCTGCTGGCACTCCACAAGATTATGAAAGCTCCGGTACATCCCTTAAGTTGGGGTGAGCAAAATTCCTGGTTACCAAACTCAAGGTGGTGAAAGCTTTATAGGATATCTATAACTATCATGTATGTAAATGTAAGCCAAGAGTGTTAAAACAGTAATTCATTCCTTGGAGTTTGGACCCCCTATGACATTTGAGTTATGCTTGTTGGATGTACAAACTATGTTCCATTACTGACTGctgcaaaataaaataaaaaaacggTTGCAAGTTAGTAACTGTTATTTTATGAA

>*HaMADS5*

TTATAttagtatatatttttttctctctctcttatTTGCTGTCAAAGTCTATTTGGTACATCTTCActgttttcttcaaaaaaaaaccCATCCTCCTCCTTCCAGAAAAGAAATGGCGAGAGAGAAGATAAAAATAAGGAAGATAGATAATATAACAGCAAGACAGGTGACTTTTTCCAAGAGAAGAAGAGGGCTTCTGAAGAAAGCTGAAGAGCTCGCCGTGCTTTGTGATGCCGATGTCGCTTTGGTCATCTTCTCAGCAACCGGAAAACTGTTTGAGTATGCAAGTTCAAGCATGCCAGAGTTGCTAGAAAAATATAAGCTTCATTCAAATAATAACGTCGTAGATAAAGTTGACGATTCACTGAACCTGCAGCTCCAGGAGAGTGATCAGACAAGGATGGGAAAAGAGTTGCTTGACAAGAATCGCGAACTAAGTCAACTACGCGGTGAGGATCTTCATGGACTTACTCTAGAGGAACTTCAGAGGTTGGAGACACTGCTGGAAGGAGGGCTTAATCGTGTGATTCGAACAAAGGATGAACGGATTTCAAACGAGATATCGACTCTTCAACAAAagGGTTTTCGGTTGATGGAAGAGAACAAGCACTTGAAACAACAAATGCTGACTTTGACTTCAAATGGCAAGAGGCCACGGACAAGGGGTGTTGAGTTGGATAATGTGGCAACTAACCCTGATGATCAAGGGCAGTCGTCGGATTCTGTCACTACTAACGTATGCAGCTGCAGCAGTGGACCTCCTCCCGAGGATGATTGTTCTGATACTGCTCTCAAACTAGCGTTACCGTTTAACTAAATTGATAAAACCGCCATGGAAGACAATGAATGAGGTTGATGGAGAGCAGGGAATGGTGGTGGTTTGATTTGAACCCAAATAAGTGTTGTATCGGctgaaatgtttaaatgacattctgaaaaacaaaaaaacacagACTTTGTAACGTGTGTTATGATGGATTCATGTGTAGTTATTTATTTAATTGCAATTTTGCACTAAAATTGCTGTTTGTTGAAAATAATCCAAACAAAGTGTTTAATTCTATTCTATTGTTGACAATGACTTGTTGAAAACTCAAA

>*HaMADS6*

ATCccttttttctctttcttctaTTATTGGCCAGCAAGCTGTGACATTTGCCACATATACACTTTCCTCTCCAAAAGTATCTCCATTTCCTTCCAGAAAGAGAAGTAGTTTGGTTCCATATCAATGGCGAGAACAAAGATAAAGATCAAAAGAATCGATAACATAACCGCACGGCAAGTGACTTTCTCAAAAAGAAGAAGAGGGCTTCTGAAGAAAGCCGAAGAACTCGCTGTTCTTTGTGATGCGGATGTTGCTCTCATCATCTTCTCAGCCACCGGAAAACTCTTTGAGTATGCAAGTTCTAGCATGCCCGAAATGCTAGGAAGGTATAAGCTTCATTCCGATAAGAGTCGCGATAAAGTAGACGAGCCTTCTCTCAACCTGCAGCTAGTAGAAAATGATGATGTGAGATTGAGCAAAGAGATCAATGACAAGAATGATGAACTCAGGCAATTAAGTGGGCAGGACCTTCAGGGATTAACCAtagatgaacttgaaaaattGGAGAACTTACTCCAAGGAGGCTTAGACCGTGTTATTAAAACCAAGGATGAAAAAATTGGTAACGAGATATTCAACCTCCAAATGAAGGGTGCTAAGTTGATGGAAGAAAACAAGCTACTCAAAGAACAAATGACGATACTTTCAAATGGAAAGAAGCCACGATCTATGTGTACTGATGCCGGTAACTTAACGAGTAACCCCGAAGATCAAGGCCAGTCATCGGAATCGGTAACTACTAATGTATGCAGTTGCAATAGTGGTCCTCCCCCTGAGGATGATGGCTCGATTACTTCACTTAAACTCGGGTTGCCATTTAACTAATGTTGAAAAAGGCAAAGATGAGTTGATATTCGTACCAAGGGCTTGAGCATATGAACGTCACACTCCCATATGTCGGGGAGTTTGTTTTTATTCATACCATTTATGAACAATTTCTCAGTCTATCACAGTTATGTTCTCTATCCAGACATGTTTAAACCAAGATATACTTTTCGTTTATTGCAGTAAGAAACATAATATTAATATGTTGAGTTTTGGATTATTAGCA

>*HaMADS7*

AAGGAAATTCTACTCATGGCTTTCAACTTGAGTTTTCATTTCATGACAGAATATATTTTCTCTCTCATCCTTTCCAGCATGCTTAAAACACTCGAGAGGTATCAAAAATGCAACTATGAAGCGCCTGAGTCGAATCATACTGCAAGGGAGGCGCTGGAGCTAAGTAGTCAGCAGGAGTATATGAAACTTAAGGCGAAGTATGAAGCACTACAACGATCCCAAAGGAATCTTCTGGGTGAGGATCTTGGCCCTTTAAACTGCAAGGAGCTTGAATCGCTAGAGAGGCAGCTCGACACATCTCTCAAGCATATCAGATCAGCGCGgACGCAGCTCATGTTGGACACATTAACAGATCTCCAAAAGAAGGAGCATGCACTAAATGAAGCAAACAGCACACTGAAGCAAAGGTTGATAGAAGGAAATCAAATAAACTCACTCCACTGGTTTCAACACGCCGAGCAACAAGTCTGTTATGACAGACAACCTGCACCTCCACAACAAAGCGAAGAAACCTTCTTTCACCCGCTCGACTGTGGGCCCACGTTACACATAGGGTACCAAATGGATCCATTAACAACGACAGAAGCAGGGCCGAGTATGAATAATTACATGCAAGGATGGTTGCCATGTTGAAAGGTAATAACACAATGTTGATGAGGTTGTGAGCATAAATTATAAAGATTCAAGGTGTGTTTAATCTTTATCGTAAGTGATTTGCGACAAACAAATATTGACAGATctatatgtatatttataaaaaCTGTTGTCTGTTTTGATGACAATTTCATGTTCTGCCAGTTTAAGAAACGGTTCAATTGTAACTTACTTTTAAAA

>*HaMADS8*

CAAAAAATCTTTAAATATTAAAGAAACAACACATCCAACAACTTTGACACTCCGTTTACCAATCtagagagagaatctagagagagaaacaccATTCCATTGCCATGGGTAGAGGAAGAGTAGAGCTGAAGCGAATAGAGAACAAGATAAACCGGCAGGTCACCTTCTCGAAACGTCGAAACGGTTTGCTTAAAAAAGCTTACGAACTATCCGTCCTCTGCGATGCCGAAGTCGGCCTCATCATCTTCTCCAGCCGCGACAAACTCTACGAGTTTGGCAGCGTCGGTGTCATGAAAACCCTTGAACGTTATCAACGTTGCTGCTTTAATCCTCAAGACAACAACAATGAACGTGAAACACAGagttGGTATCAAGAGGTTTCCAAGCTAAAAGCCAAGTTCGAATCGCTTCAGCGCACACAAAGACATTTACTTGGGGAAGATCTTGGACCACTTAGTGTCAAGGAACTacataatcttgaaaaacaactTGAGGGGGCTCTTACTCAAGCTAGACAAAGAAAGACACAAATCTTGGTAGAACAGATGGAAGAGCTTCGCCGCAAGGAACGCGAACTTGGAGACATAAACAAGCATCTAAGGATCAAGGTCTCACACGAGATGACAACGTTTGAGACAGATCAAGGTCAGGGCTATAGGGCACAACTTCCATGCCCATGGAACTCTGGTGTATCTcctggtaacaacaacaacacatTCCCAATGCACCAATCTCACTCCAATCCTATGGATTGTCAACAAGAACCCATCTTGCAAATAGGGTATAACCAGTTTATGCATGAGGAAGGACCATCAGTTCAGAGGAGCATGGTTGGAGAGAGTAGTATGCAAGGCTGGGTTAATCTTTGACCATTATTCTATGTAAGGTCTTCTACGTCAGCAGCATCTTATTTGTAATAATATTGATGAAGACTTATTTGGTAGGATGTTTTTGTGATAATTTGCAATGTGTTTGCTAACATTAATTATAATATAAGGGTGTAATATTATATACATATTCAGATATTCTAATATATGCATCTATATGATat

>*HaMADS9*

TACCTCTGTGCAATGATGTGCTATTATTCTTAGGCTTATAAACTGTTTCGTTTTTACTATTTTTTCACTCCCCTACCCCCATAGTCCCTTCTTTCTTTATTCAAGCACTGTACgtcctctttctttttcttctacaTCTCTCTCCTTAGCAATCCAAATCATTCCCAAAGAAAGAAAGGGTTGTTTTACATCTCTTGGGTTTGAAAATGGTGAGAGGGAAGACTCAGATGAGAAGGATAGAGAATGCTACAAGCAGACAGGTGACATTCTCCAAGAGAAGAAATGGTTTGCTAAAGAAAGCTTTCGAGCTTTCTGTGCTTTGTGATGCTGAAGTTGCTCTCATCATCTTCTCCCCAAATAGCAAACTTTATGAATTTGCAAGCTCAAGCATGAGCGGTACTATCAAACGTTATCGGGAGCATGTAAAGGAGAATCAAACTCCGAATTCTGCGTGTCCAGAAGATGTCCAGCATTTGAGGCAGTTAGCTGAAGGCATGGCAAAGCAGATAGAACTCTTAGAAGTTGCAAAAAGGAAAATTTTGGGACAATGTCTTGGATCAACCACCATTGAAGAACTACTACCGATTGAAGAACAGTTGGAGAGGAGTGTATGCGTTATTAGAGCAAGAAAGATGCAAGTGTACAATAAACAGGTTCAACAATTACAATCAAAGGAGAGAATGCTCACAACTGAAAATGCAGTGTTAAATGAGAAGTATCAACTTCAAACTACTGAAGAATCAGAAGAAGGGATAGAGAATGTCATAGTCATGGAAAACAAGGATGCATCAGATGTGGAAACAGGTTTATTTATTGGGTTACCAAAGAGGAACATcaagaacaattgaagaaagtgaTATTATGATAATATTAGCATACGCGTTAGCTTAAAAAATGATCGCTATTTAAAGAAGCTTCCTTCCTATATGTAGCAAAGCAGGCTTTCTATGTTTTTATATGGGGGTGGTTTCATAAGCATTAAGCATGTATGATGTACGTTATGTAtgcatatacatatataatatgATTGTTAAAAGTAAGTTGATATCATTGTTTGAA

>*HaMADS10*

CCACTTTGGTTTAAATTCCATCAAAAGATGTACACCAACACCaaccactctctctctctctctcgctatACAGATTTCATCAAATAGTGAACATACACCAGAATAGAAACTAAAACATTCTTCTGCTTCTTTACTTCTTGCAAAACATACATACCAAAGTCTTGTTCTTTCTTTttagggttttctgtaaaaaCCCAAGAAGAATAATAACAAGTACTTACCATATCAAGATCAAGATCAAGATCAAGGGATCATTGTTCCGACAAAAAACCAAAAAGAAggttttgatcaGTAAGAAAATGGGGAGAGGGAGGGTACAGTTGAAGAGGATTGAGAACAAGATTAGTAGACAGGTGACATTCTCCAAAAGAAGAACAGGCTTGTTGAAGAAAGCTCATGAGATCTCAGTGTTATGTGATGCTGATCTTGCTCTTATTGTTTTCTCTCCTAGAGGCAAACTCTTTGAGTTCTCTACTCACTCCAGTATGGAGGCAATACTTGAAAGATATGAACGATACTCATATGAAGAAAGGCAGCTTACTGAATCTGAATTAGAAACACAGGGACGCTGGAATCTTGAATCCTCCAAGCTAAGAGCGAAGATTGAGGTCTTAGAAAAGAACATAAGGAACTATGTCGGGGAAGACCTCGAGCCCTTGAACCTTAGAGAGCTTCAAAGTATAGAACAACAACTTGAGACTGCTCTCAAACGAGTAAGAACAAGAAAGAACCAAGCCATGCATGAGTCTATCTCAGAACTCCATAAAAAGGAGAGAGCACTCCAAGAGCAAAACAGTGCACTCTCGAAGAAGTTGAAGGAGAATGAGATGAACGAAGAGCAGCAAAATACTCAAGATCATACAATCTCTAAGGCAATGTTGCCCCCATGGTTGCTTCGCCACATGAACGAACAATATGAGATTTAAGTAAGTATGCAAGTCCTACAGTTGCAGTGGTTATGTAATGTTTCGTATTTGGATTCCTATCATAACGTATGTTGTTGGGTTTTGAAGGCTTTAATCACACAGAGGAAACTACGTACAGAGAATGTGTttataaaaccctaatttcattcatgCTAAAACTTACATTATATAGAATAAAATACATACCCACTAACTAATAATTAGGGCCACTAACCCATTAACCCCACTATAACTTAATCTTGCTAAACACTTCAACATAAAAAACACATGACTAATTAAAATACTTGACCCATAAAAAACACACTTTTACCGACCCGACTTGACCCGTCAAGATTAATCTAacattcacccccttaatcttgacattttgacccgtttcttTCTTGCTCGTTGATTGAACCACATAGGTTCCGATCTTGCTCTTGCCATACGAACCGTTAAGGTTCgattctttctttcttttggcTACCGATATTCTTTTTTCTTTCTAATCGACgccctttctttctttc

>*HaMADS11*

ACCAACCACACACACTCCTATCTCTCTCTCTGAATTATGCTTTCTTCATCCTACCAAAACCACTCTGGTTTTAAGATCATCAAACCTTTTTGAACATAGTATACCAACCACTCAGTCTactttatctctctctctctctcacccaaATTATGCAATTTTCCTCCAAACACAAATAACAGATTTCTTCATCAAATTATGGATATACACCAAAATAGAAACTAAAACATTCTTCTGCTGCTTCTTCAATTATTGTAAACATACAAACATACCACAGTCTTGTTGATTCTCTTTAGGGTTTCTTGTAGAAACCCAAGAATAAAAACAAGTACTTACCAGATCAAGATCAAGAGAcctcaaaacaagaaattaaGggTTTTCAACAAGAAAATGGGGAGAGGGAGGGTGCAGTTGAAGAGGATTGAGAACAAGATTAGCAGACAGGTGACATTTTCTAAGAGAAGAACAGGCTTGTTGAAGAAAGCTCATGAGATCTCTGTGCTATGTGATGCTGATGTTGCTCTTATTGTTTTCTCTACTAAAGGCAAACTGTTTGAGTACTCCACTCACTCCAGTATGGAGGCAATTCTTGAAAGGTATGAACGATACTCGTATGCAGAAAAGCTGCTTACTGGACCTGAAGCAGAAACACAGGGAAGTTGGACTCTTGAGTCATCCAAGCTCAGGACTAAGATTGAAGTACTAGAAAAGAACATAAGGCACTATGGTGGGGAAGACCTTGAGCCTTTGAACCTTAGAGAGCTTCAAAGCGTTGAACAACAACTTGATACCGCTCTCAAACGAATTCGAACAAGAAAGAACCAAGTTATGCATGAGTCCATCTCAGAACTCCATAAAAAGGAGAGAGCACTCCAAGAGCAAAACAATGCACTCTCCAAGAAGTTGAAGGAGAATGAGAAGAACACCGAGCAACAGCATGTAGGGCTTCTGTTATCTCAGCCtcagccgccaccaccaccaccaccgcccccGCCATCTCAACCACCACACTTAGTTCCTCTTGCAATTGGGAGTACTGGACCATTCCAAGGAACAGCGGCTACAAGGGCGGATGATTCGGCTCAAATCCATGCCTTATCTGCAGGGATGATGCCCCCATGGCTGTATCGCCACGTCAACCAATAAGACAATGGCATTCTAGTATATTTATGAAGTGGCATAAATGAGTAATCACTTATGTACTTCTTCATGTCTGGATCCCTATCATAATGTATGTGCAATGATCACTGCTACATAGACGGTTATCGTTCAGTTTCTACCCACTACATTAATAGATGTTCCTGCATGATATTTTAAAGATACAACCAATTAACATGTGATGGTTTTCGTCA

>*HaMADS12*

ACCAATGGTAGATGGAGGTTGCCCAAAGATAGATCACCCCAGGGTTTCCATTTTCTTCACACATTTCTCTTTTGAGAACTCATTTCTCTCTCTCCCCCCTCTTATTTTATCTATATTCAAAACACTAGTTTCATGAATTAAAAGAACTTCTAAAAGTGGGTGTTCTCTTAACCAAGAGTAGAATTGTTGCAGAAAGgaaaaaaacacaaataaaaaacatctttttttactaataatcatGGGAAGAGGGAGAGTAGAACTGAAGAGGATAGAGAACAAAATTAACAGGCAAGTTACTTTTGCTAAGAGAAGAAATGGGCTTCTCAAAAAAGCTTATGAACTCTCAGTTCTTTGTGATGCTGAAGTTGCTCTTATCATCTTCTCTAATCGTGGCAAGCTATATGAGTTTTGTAGCAGCTCTAGCATGGTGAAAACCCTTGAGAAGTACCACAGCTGTAGTTTTGGATCATTGAAAGCCTCCCAACCAGAAAATGAGAGCCAGTACAATTACCATGAATACCTAAGGCTAAAGGCAAGAGTGGAGGTTCTGCAACGGTCTCAAAGAAATCTTCTTGGCGAAGATTTGGCCCCATTGAACACTAAGGAGCTAGAGCAACTTGAGCATCAACTGGAGACTTCTTTGAGGAAAATCAGATCAACTAAGACTCAATCCATGTTGAATCAGCTTGCTGAGCTACAAAGAAAGGAGCAAGTTCTTGCTGAAACAAATAAAGCCTTAAGGAATAAGCTTGAAGAAAATGCTCAGGAGTTTCCAAGGCAAATGTGGGAAGCTAATGCACAAACAATCCCCTATAATCCCCTTCCTACACACTCTGATGAGTTCTTCCAGCCTTTAGGATTGAACACCGCCATGCACAACAGTTTCAATGGATTGAGATACAACCCTATCGGCTCGGATGAGATGAATGTTGCTGGTGTCAATGCCAACAATCATAATGGATTGTTTCCGGGTTGGATGCTGTAGTTGAAGAAATGTGTAACCCTAATTTTCGCAAGGGTAAAAATAAGCGATCGAAGACTTCATAAGTCGTTGAGAGGACGACGATGCCTTCGATTTGAACACACTATGGATTATTGGCATGTATGTCCTTAATTGCATAAGATTTGAAGTATTTTGGATTTGTGAGATAATTATTTTTGTTTACTATA

>*HaMADS13*

AGGAAATCCCACAAGTTCACTATTATATATTGGTTGTGTTTTATAAGGTTCATGTTCGCTCCAACTTGCAACCCAAATTAAAAGAAATATTGGTTGTGTTTTCTATAAACACGTGCTAAAATGATTTGCAGGCTAGGGATAAGAACGATCTGAAGTTATAAGTACGGCTGTGATCAAAACACGTTTTACATAAAATTAAAAGAGCCGACAAAACATGTTTCCGTTGTTGACCACTGTGATAACTGGTAACATAAATAAGAGCGATGTATGTATATGTGGATAGAGATCTAGATAAGAGCTACTGTTGAAATTTAGCGACATCTCACCACCTAGATCGCCGGAAGAAAGAAGAATGGGGCGGAGGAAGCTCGAAATAAAGCGGATCGAAGACAAGAGTAGCAGGTTAGTCACTTTCTCCAAGAGGAGATCAGGGTTGTTTAAGAAAGCTCGCCATCTTTCCGTCCTCTGCGACGCCGATGTCGCCGTCATCGTCTTCTCCGCAAGAGGGAAGCTCTATGAGTTTTCTAGCGGCAGTAGTAACAGCGTGCAACGTACTCTTTCCAGATATCAGAaaagatgttctgaagaaaaagAGATAAACACCAACGGAGTGGGAGAAGATTTATGCAAAAGATTTCGAACATGTAAAGAACTTCTACAGACAGTGGACAGGCTTGCTGAATTACAGGATAATGCTGAGGAACTCTCTGTGAATGACATGATACAACTAGAACAAGAACTTGATTGTGCTCTCATGCAAACACGATTAAGAAAGGCACAACTAATGATGGAGTACATATCAACCCTTAAAGAAAAGGAAAGTAAGCTACGTGAAGAAAACGAAAAATTGGAAAAGCAGGTTGCCTCAACACAGGGAAATGATGTTGATGATGGAGGTGGTGGTCTTAACGAACTTGCTACTAACCAGATCAACCCACCCAAACTTACAACACTCCCTCTTTTTAATGGCTAAGATCTGTGATCAAAAGGGCCAATAATCCAACTGTCATTTTATGGTTGTGAAAGAAGCCTCATCTGCCTTGTGTTTATGTAATCTTTTTGTG

>*HaMADS14*

TCCTTCTTTGTTTGCCGTATAGACAGAGGGAATACAAGATAATACATGAATAAGGAAATTACCCTACCCAGCTGAATTTGTGGTCTGAAAGTAAAGGGGTCTTTGCATCACCTACCAAATATAATCCTCCATGTctctttttcttgttttgacTTTCTTCGCTAGATTTACATGAACATCTAGTACTTCTTTGCCCAGATCTTGATCGCAAGTCTCACCTTTTGTGTTCTTCTTGTTTAATCTTGGAAATGGTGAGAGGAAAAGTTGAGCTGAAAAGAATTGAGAACACAACAAGCAGGCAAGTGACCTTCACTAAGAGAAGAAATGGGCTTTTGAAGAAGGCTTATGAGCTTTCAGTTCTTTGTGATGCTGAGGTTGCCATGATAATCTTCTCTCAAAAGGGAAAACTTTATGATTTTTCAAGTTCAAACATGCGGAAGACTATAGAGAGGTATCGTGAACATGTTAAGAAAGATGAAAACTGCATTCCTGAAACTGAAGTAAACACTCAGAAACTGAAGAAAGAATCTGCCATCATTCAACAAAAGCTAGAGCAACTCGAAGCTTCACGACGGAAGTATCTGGGACAAGACTTAGTATCATGTTCACTGGATGAAGTAATCGAGTTAGACAGTAAGCTGGAACATGCATTGAGAACCATCAGGGAAAGAAAGGCTCATTTATTTAAAGAACAAACTGAAAAACTGAAAGCAAAGGAAAGATACCTCATGGAAGAAAATGCAAGATTAGTGCAAGAAACTGCAATTTTATGCCAAAAGCGTGATAGAATTTCACCCCAGCATTATGTGAAGGAGAAAGATGTGATTACAAGAAGCCAAAGTAGCCCGTTTTCGGAGGTGGAAACAGACTTGTTTGTTGGCCTGCGCCCCTCTCATAATTATCGAGATTAATAGTTCTATAGTTTTGCTGATAAGAATGATTAGTTATCTGACTTGATCTATTCATCAAAATTCCTGCAAACAACTTGGGATACAAAGAATAAAAAGTTAATAGCAATTAAAGGAAGTGCACATGTACTTATGTAATAAAACCATAACTGTAGTTTTACCAGTTTCCTTTGTATGGTAGTTACCACTCCAGTTGGTTATGACAACTATGCCAATTGGATGATGCATTAATGTTACACAGAAGATGGTTGTGTTATCTA

>*HaMADS15*

AAGGCATACACCAAAATAGCAACAGAAAAAAAGACATTCTCCTGTGTCTTTCTCGTTATCGTCTTCTtagatcttcttcttcttctccttttTTCCTTCTTTTTTTCACTTTAAAAGTTGGTTTTTTAGGGTTTTCTGTAGAAAGAAAACTGTAAACAGATGGGGAGAGGTAGAGTGCAGTTGAAGCGGATCGAGAACAAAATTAGCCGACAAGTAACATTCTCCAAACGAAGAACTGGTTTCCTCAAAAAAGCTCATGAGATCTCTGTCTTGTGTGATGCTGAGGTTGCTCTCATCGTTTTTTCTTCAAAAGGAAAACTGTTTGAGTACTCAACTCACTCCAGTATGGACACTATTCTTGAAAGGTATGAAAGGTATGCTTATGCAGAGAAAATGCTAACTGCTCCTGAAACAGAAACACAGGCGAGCTGGACTCTGGAATCCTCTAAGCTCAAGGCAAagattgaagttcttgaaagaaACATAAGGCACTATGTTGGGGAAGACCTGGAGCAGTTAAACCTAAGAGAGCTTCAATATGTAGAGCAACAGCTCGAAACAGCTCTTAAGCGGATACGAACAAAGAAGAATCAACTAATGCACGAGTCCATCTCCGAGCTGCATAAAAAGGAGAAGGAATTGCGAGACAAAAACAACTCACTCTCTAAGAAGTTAAAAGAGAATGAGAAGAACAGTGAGCAACAAGATTCAATGCTGCAGGTGCTTCAGCCACATTCAGTTCCTTCTTTTGCAACCACCagCGCACCAATATTCATGGGAGCAGCCATGAGAGAGGAGGAATTAGCTCAAACTCATTCGGTATCCACTAGCATGATACCACTTTGGATGATCCGCCACATTAACCAATAAGATTATGATCTCCAATATATAGTAGTCCAGTCTGGGTTTGAACAATGATGATGTTCAAATTGCTGCTACATAAATATATAAACCCTTATGGGTTCTAGTATTCTCTATGTTCAAACACTGGAAATGCATCTAATATGACCTAAAtgatgatataatatcagtaaaaGACGTGTGTTGTTACGTTGATATATATATGAATGTATACATGGGGTGAGAATGCATTCTGAAACATGTATGGATTGATATTAGATTCA

>*HaMADS16*

CacacacactcagTGCTTATAGGGCACACCATGATGAGATGCACATAAACAAAATAATCATTCGATCGGTTGAACTCTTTCTCAGAAACTGGTGCTCTATCTTCTTCCAAGGAAGAAAAGATGGGGAGAGGAAAAATTGAGATCAAGAGAATTGAAAACAATACGCATCGGCAAGTCACCTTCTGCAAGAGGAGAAACGGGCTGCTAAAGAAGGCTTACGAGCTTTCAGTTCTTTGTGACGCTGAAATTACGCTAATCGTCTTCTCTAGCCGTGGAAGACTCTATGAGTACGCCAATAACAACATAAAATCAACCATAGAGAGATACAAGAAGGCTACTTCAAGTACACCAGACACATGGTCAACTCAAGAGGTCAATGCTCAATTCTATAAGCAAGAGTCAAAGAAACTTCGCCAGCAGATTCAAATGCTTCAGAATACTAACAGGCATCTCATGGGTGACGGATTGGAGCACTTAAATGTGAAGGAACTGAAGCAATTGGAGGGCAGGCTTGAAAAGGGAATCTCAAGGATTAGGTCCAAAAGGCATGACCTGATACTAGCTGAAACTGAAAATTTGGAGAAAAGGGAGATCGAGCTCGAACATCACAATGCATTCCTACGTTCAAAGGTGCAGGTTGCTGAGAGCATGCAGCAGCTAAACATGAATACTGGGGAGGACTATAATGCATGGCAAGCATACATGGCTAGGAACATGCTTCACCTCAATATAATGGAACCTATGACTCTGGAGGCTAGTCCTTCAACCTTCTCCATTTGTCCCAAGCCTTCTCTTCACCTTGGGTGATGTCGCAAAGGGAGATGACCACGACAATGATGCAGTGGTAGCTTATTATTGTGTTGCTTTTGGAATAAAGATCTTATGGATGCCCATGTATGTTTGGCGAATGACGTAATTAAACTTAACtatgttgtgtgtgtttttcatTTGTGATGTTATCAAATGTTTTAATTTGAATGAAGTTTGCAAACTTATATATCA

>*HaMADS17*

CacacttgatCATCTTCTACATCTCTCTTCATATCCACCAGAAACTTAACTTCTGCTTCTTCAGATGGCAAGGGGAAAGATCCAGATCAGGAAGATCGAGAACTCAACGAACAGGCAGGTGACGTATTCCAAGAGAAGAAACGGACTGTTCAAGAAGGCGAATGAGCTCACAGTGTTGTGTGATGCTAAAGTCTCCATTATCATGGTGTCTTGCACTGATAAGCTTCATGAATATATAAGCCCTTCTGTTACGACGAAGCAGTTTTTTGATCATTATCAGAAGGCATCTGGAATTGATCTTTGGAACTCTCATTATGAGAAAATGCAAGAGGAAATGAGACAACTGAAAGAAGTGAACAAAAATCTTAGAAGGCAAATTCGGCAAAGGCTGGGTGATTGCTTGGAAAATCTAGGTTTTGAACAACTGCTTGATCTTGAGAAGGAGTCACAGGAGGCTGTATACATCATTCGCGAACGCAAGCTCAAAGTGATTGGTAATAAGTTGGAGACTTTGAAGAAGAAGGTGAGGAGTGCTCAAGACGTATACAAAAAGCTCATGCATGAATTTGATATTCGAGGAGATGATCCACAGTACGGGATGATAGAAGACGCAGGAGAGTATGAAGCCATGTATGGATACCCGCCACATATCGCTTCCACGCCGCAGATCCTAACTCTACGGCTGCAGCCTAATCATCCAGAAAATCTTCATGCTGTTGCTTCAGACCTCACCACCTATGCCTTGCTTGGCTAGCTAGCTAGTTCCATTGTACCAAACCTATAATTAAGCCCTTGGAAGACATTAAGTAGTTGGTGTTTGATTTCTGTGCATTGTGATTGGTAATGTGTGAAGTTAATACTTGATTAATTGTTGTTGTtttatgttttaataatactTAATTGTGacatgttta

>*HaMADS18*

TTAAGAAGTAAATGAATTGATAAGTACAAGAGATAATATGGAGATGTCTTGGACATGTCAAAACCCCACAAGGAAAGACACTCTTGCTCACTCTTTTGGAAAGTCTCTTGTTTTCCAGCTCTAATTAGAAATCaatctctctctcacacacacacacatacacacacactttGTAATTCTATTTAATAACTTTTGTATCAAGATTCATTATTGTTAACTGAAAGCAAATTGAGAGAGGGGAGGGGATGGGGAGAGGAAAGATAGAGATCAAGAGGATAGAGAACACAAGTAACAGGCAGGTCACTTACTCTAAAAGAAAGAATGGAATCATCAAGAAAGCTAAAGAAATTACTGTTCTGTGTGATGCTAATGTCTCTCTTGTTATCTATGGCTCTTCTGGCAAGATGTATGAGTACTGCAGCCCCAAAACCAACTTGATTGACATGCTGGATCGGTATCAAAGGCTTTCTGGAAATAAGTTGTGGGATGCTAAACATGAGAATCTGCAGAATGAaattgacagaatcaagaaagaGAACGAGAGCATGCAAATTGAGCTCCGGCACCTGAAAGGGGAAGACATAACTTCTTTGAACTATGAAGAACTAATTGGATATGAAGATGCACTTGAAAATGGTCTGACCAACATTCGTGAGAAAAAGGATGAAATCCCCAAAATCATGCGAAAACGCGAACAAGTTCTTGAGGAGGAGAATAAGCATCTCATGTATTTGGTCCAACAAAGTGAAATGGCAGCCATGGGAGATTACCAACAACATGAACCCTTTTCATTCCGTGTCCAACCAATGCAGCCAAACTTGCATGAGAGGATGTAGTAATATATGCATTTATGCAAATCATGTTGCGATAAGGTTTGTAATACATTAACTATGGATGTGTTTTGGTAGGTGTGTCGTCAATAATGGTGCGCTCTTGGGCGTGTGTTGTTTCTTGTAACTTTGTGTTGTGAACTCATGATGATGGTCATGTATCCAAATTggtatctatctatctatataaCCAAGTATGTAAGCGTGATTTATAACACTTGGA

>*HaMADS19*

TCCTATTTCTAGTAACAATTCCACCTTCCTTTCTTCCTGTTCCTTTCCCTTCCCCACACACACTTACTGTGTGTTTTCTCCCCACCACACATTCATTCTCAAACACAACAAACTCATCTTCTCAAACCAATCACTTACAGATCAATAACAActtctttttaacaaaaaaacATCCAAAATCAATGGCGAGAGGCAAAATCCAAATTAAGAAGATCGAAAACTCGACGAACAGGCAGGTAACTTATTCGAAGAGGAGGAACGGGTTGTTTAAGAAGGCGAGTGAGCTCACTGTGTTGTGTGATGCAAAAGTCTCCATTATTATGGTTTCCTGTACTGATAAGCTTCATGAATACATCAGTCCTTCCATTACGATGAAGCAGTTTTTTGATCAGTATCAGAAGGCATCTGGAGTGGATCTTTGGAACTCTCATTATCAGAAAATGCAAGATGAACTGAGACAGCAGAAAGAGGTGAACAGAAATCTTAGGAAGCAAATTAGGCAAAGATTGGGTGATTGCTTGGAAGATTTAAGTTTTGAAGAATTGCTTGCACTTGAAAAAGATTCACAGGAGGCTGTTTATGTTATTCGTGAGCGCAAgCTCAAAGTAATTGGTAATAAAGTGGAGACTTCAAAGAAGAAGgtGAGGAGTGCCCAAGATGTCTACAAAAAACTCATGCATGAATTTGACATAAGAGGAGAGGATCCACAATACGGATTGATAGAAAATGGAATAGAATATGACAATGTATATGGATACCCGCAAATGGGTGCACCACCACATATTCTGACTCTACGGTTGCAGCCGGACCATCCCAACAATCTTCATGGTTCGGTCACCACCACTGGACCCACGGCATCTGATCTCACCACATATGGCTTGCTTGGTTAGCTATTAGTTAATAACTAAAGTTCAATTTTAAGACAAATCAATTGTGTGTCGACTTGTTCATAAGGTTTTGCCTCGTTTGATTGGCTTATGATATTATCACTTTGTGTGGTTACTTTTTATGTTTTCAAGAACTAGTACTCTAGTGGTAATTCACATATTCGCATATatggtgtttgatttgtttata

>*HaMADS20*

GTCAAAACCCCACAAGTAAACACACTTTTGTTCACTCTTTTGAAGGAAAGTCTCTTGTTTTCCAGCCATTATTagaaatctctctctctctctctctctctagaattctaTTAATTAAGTTTTGTAAGAAGATTCATTACATCTCTCCCATTGTTATCTGAAAGCAACTTAAGAGAGAGAATGGGAAGAGGAAAGATAGAGATCAAGAGGATAGAGAACACAAACAACAGGCAAGTAACATACTCCAAAAGAAAGAATGGAATCATCAAAAAAGCTAAGGAGATTACTGTTCTCTGTGATGCTAATGTCTCTCTTGTTGTATATGGATCTTCTGGCAAGATGTATGAGTACTGCAGCCCCAATACCAACTTGACTGACATGCTGGATAGATATCAAAGGCTTTCTGGAAATAAGTTGTGGGATGCTAAACATGAGAATTTGCAGATTGAaattgacagaatcaagaaagaGAATGAGAGCATGCAAATTGAGCTCAgGCACTTGAAAGGGGAAGATATAACATCTTTGAACTATGAAGAACTAATTGCATTTGAAGATGCACTTGAAAATGGACTTACCCACATTCGTGAGAAAAAGGATGAAATCCCCCAAATTATGAGGAAGCATGAACAAGTTCTAGAGGAGGAGAATAAGCACCTAATGTATATGGTGCAACAAAGTGAAATGGCAGCAATGGGAGATTACCAAGCAGATGGACCCTTTTCTTTCCGTGTCCAACCGATGCAGCCGAACTTGCATGAGAGGATGTAGTCACATTTTCTGTATGCAAGCACGTCTTGACCAATTGAAGCTTATAATTGCATTAATTACCTGAGGTTTGTAATCCGTTAAGTATGGAGATATGGTGATTTGCTAGGACTGCTATGTTTCTTGTAGCTTTATGTTTTAAGATCATCATGATGGTCCACCTATTAAAATTGCGGGTGTGCCTTGTTGGAATTATTAAACTAAGTTATGTTTATGTATGTTAA

>*HaMADS21*

AAAATATTAGGTATTTAGATCAAGAAGTGTTTTAAAAAAGTTTGTTAATGTCATGGACAGGAGTCATTGTCCACTTTATAAAAACTCATCTTCTACCCACCTCTCCTTCCAGTTTTTGCACCTTTCCTGCTTGGTTTTGAAGGAAGGCACTCAAAAAAGCAAAAAAGAGAGAAATTTTGAAACTTGATTAGTTTCTCTCCTCAATTATACATAAAGATTCATCCCTCAACCCCACTCATTTATTTCTTTTGTGCACTAGTAACTATCAGAAAACCAATCTGAAAATCAAGTTTAGTTTCTTCCCAAATTTCTTGAGGTGAAGTACATGTAATTTACTTCAGGATTCATGGAAAATTCTGAGGCTATTGAGCATGATTTCAGCTGCATCGATCGATCCATGTCGTTTCAAAACGAGGACTCGGGGGACATATCTCCACAGAGAAGGATTGGGAAGGGAAAGATCGAGATCAAGCGGATCGAGAACACTACGAACCGACAAGTGACTTTCTGCAAGCGTCGTAATGGGTTGCTCAAGAAGGCCTATGAGTTGTCTGTTCTTTGTGACGCCGAGGTTGCTCTTATCGTCTTTTCGAGCCGTGGCCGTCTTTATGAGTATGCTAACAACAGTGTTAGAGGAACAATTGATAGGTACAAAAAATCATGCTTAGATCCACCTAGCACGGGTTCCGTTGCTGAAGCCAATGCTCAGTTTTACCAACAAGAAGCTACCAAACTGCGTCAGCAAATTGCAAACCTTCAGAATCAAAACAGGCAATTTTACAGGAACATCATGGGTGAATCTTTGGCAGATATGCCGGGGAAGGAGCTTAAGAACCTTGAAAGTAAGTTAGAGAAAGCAATTAACAGAATTCGTGCGAAAAAGAATGAACTATTATTTGCTGAAATTGAGTACATGCAGAAAAGGGAACTTGAGCTGCATAACAGCAATCAATTCCTTAGAGCAAGGATAGCTGAAAATGAAAGAGCTCAGCAGCAGCACATGAGCTTGATGCCTGGAAGTTCTGGTTATAATGACCTTGGACCACACCAGTCCTTCGATGGTCTAAACGATCTTCAAACGAATGAGTTGCAACTCAATAACAACTACTCTTGCCAGGACCAAACCCCTCTCCAGTTGGTGTGATAACGACATGGAGTTCTCCATTACTCGTTCAACCGGAGCTATAATAACTTGTTTATGATTAATTGACAAAGACTAGACTTATATTCTTATATTAACTTGCTTGATTTAGTACGGATTTAGTTCATGGCTATCTAAGCCAGAGAGCTCTAAATATATATGTGTACTTTTATGATTATGTATGTGTTTTAATATTATGAAGCAGCTTATATGTTA

>*HaMADS22*

ttgcTATAAGTCTTGAACAATATATATAGCAAAAAAATGATTGAATATTTGGTATTAAGATATAATGATTAAGGTTAAGAGCGTTTATTAATTTAAAGGACAAAAAAGTCGTTGTCTACTTTATAAATACTCATCAACCATCCCACCTCCCCATTCCATTTTCTGCAATCTTTCCTGTTCAGATTTGTGGGAAACCCTCAAAAAGCAGAGAGAGATCAAAAAGGTGTATCCATGTCTTTTCCCAATGAGTCAGGTGAGATGTCTCCACAGAGAAAATTGGGTAGGGGAAAGATCGAAATCAAACGGATCGAAAACACGACAAATCGACAAGTGACATTTTGCAAACGCCGCAACGGTTTGCTGAAGAAGGCTTATGAGTTGTCTGTACTTTGTGATGCTGAGGTTGCGCTTATCGTCTTCTCGAGCCGCGGTCGCCTCTATGAGTATGCCAACAATAGTGTGAAAGGAACCATAGATAGGTACAAAAAGGCATGCCTGGATCCACCAAGCAGTGGCTCTGTTGCAGAAGCAAATGCTCAGTTTTACCAACAAGAAGCTGCAAAACTCCGTCAGCAAATCGCCAACCTTCAGAACCAAAACAGGCAATTTTATAGGAACATCATGGGTGAATCACTAGGAAACATGCCAGCAAAAGATCTCAAGAATCTTGAAGGCAAGTTAGAGAAAGGCATTAGCAGGATCAGATCAAAAAAGAATGAACTTCTGTTTGCTGAAATCGAGTATATGCAAAAGCGAGAAAATGAGTTGCATAACAATAATCAGTTCCTCCGAGCAAAGATTGCTGAAAATGAAAGATCCCAGCAGCAACACATGAGCTTGATGCCTGGAAGTTCTGATTATGATCTTGTACCACCTCATCAACCATTTGATGGTCGAAACTACCTTCAAGTCAACGATCTTCAACCGAATAACAGTTACTCTTGCCAAGATCAAACCCCTCTCCAGTTGGTGTAATTCATGATGGGCATAAAGGATTTGAGGTTTCTACTTGCTATAACATCTTCATGGTGATTAATATGGTTTTATGGTTTGTTACAACCTTATTCTAAGTATCTAGTTTGACTTTTGTGTTCTTGTTTTATTTGCCATGGGTGCCAACAATGTGTATTTTATGAAACAACTTGTTTTTCCTCGGGTTATCTTCTTTGTATTATATGCTCAAGGATTTCTTGTGAGTA

>*HaMADS23*

TcactttcttcttGTACCCCTTCCCTTCTCTCTcctaatctctctctctctcgatcttCATCTATATCTTTATTACTTCATATCCACCACCATAAGTTCATCCTCAGATCGATCGGAGGTGCCAACAACATAGGGGATGGCGAGGGGAAAGATCCAGATCAAGAAGATTGAGAACTCCACCAACAGGCAGGTGACGTATTCCAAAAGAAGGAACGGATTGTTCAAGAAAGCGAATGAGCTGACGGTGTTATGTGATGCTAAAGTCTCCATTATCATGGTCTCTTGCACTGAGAAGCTTCATGAGTACATAAGCCCTTCCATTACgACGAAGCAGTTCTTTGATCAGTATCAGAAGGCGTCTGGAATTGATCTATGGAACTCTCATTATGAGAAAATGCAAGAGGAGCTGAGGCAGCTGAAAGATGTGAACACAAATCTAAGAAGGCAAATCAGACAAAGGTTGGGGGATTGCTTGGAGAATGTAGGGTTTGAAGAATTGCTTGATCTTGAGAGGGAGTCACAGGAGGCTGTATACATCATTCGCGAACGCAAGCTTAAAGTGATTAGTAATAAGTTGGAGACTTGCAAGAAGAAGGTGAGAAGTGCTCAAGACGTGTACAAAAAGCTAATGCATGAATTTGATATAAGAGGAGAGGATCCGCAATACGGGATGATAGAAGATGCTGGAGAGTATGAAGCCTTGTATGGATACCCACCACATATCGCTGCTGCACCACGAATCCTCACTCTGCGGCTGCAGCCTAATCATCCAAATGATCTTCATGCTGCTGCATCAGACCTAACCACCTATGCATTGCTTGGCTAGTACCAAACCTCTCCTATGTATTTTAAAGACTTTAATTAGTTGTATCCAAACAGTACCCCTCCACTTTTTCTGTATTGTGACTGATGATGTGTGTGGTTATTTCTTAACTAATTGTTGGTGTTTGTATTTAAA

>*HaMADS24*

GGGTGTTTGAAGGTCATTAGAGAAAAAAGTAAGGGTTCACTGAAGCATAAAAGGTAACTCCCCAAAATCACCTACCCACCCCACCCCTTATAGAAACAACAAATTAACAGCTTTCCTTTTCTAGCCAAGAGTATCTTTAGTCTCTGGATTTGGTTTGTAAGGTTTTCTGAAGATAGCTTGAGAGGGATGGGGAGAGGAAAGGTACAACTAAGGAGGATAGAGAACAAGATCAACAGGCAGGTAACTTTCTCAAAGAGGAGAGGTGGCTTATTGAAGAAAGCCCATGAAATTTCAGTTCTTTGTGATGCTGAAGTTGCTTTAATTGTCTTCTCCAACAAAGGAAAGCTCTTTGAGTTTTCTACTGACTCTTGCATGGACAGCATCTTGGAGCGATATGAGCGATATTCTTACACTGAGAGACAGCTAGTTGCAGCGGATGCTACTCCGAGAAGTTGGACCCTAGAGTACAATAAACTGAAATCTAGGGCTGAGCTTTTGCAAAGAAATCACAGGCACTATATGGGTGAGGATATTGAGTCACTGAGCTTGAAAGAAATCCAAAATCTGGAGCAACAGCTTGACACTGGTCTCAAGAACATTCGAACAAGAAAGAACCAACTCCTGCATGAATCAATCTCCGAGCTTCAGAAAAAGGGAAAGGCCATACAGGAGCAAAACACTACTTTGACAAAGAAGATCAAGGAGAAGGAGAAAGACAAGACAATACCACAAAACACTCAATGGGAGATGCACAACTATGTCGACCACGACACAACCTTCCTCATGCCGCCCCCACCTCCCGCTCTCAATATGGGGGGTGATTACAAccatggtggtggtggtagtagtGAAGGAGCTGATGGGAGGACCAATGAGCTTGACCTAAGTCTGCAGCCAATATATTCTTGCCACATGAGGTGCTTTCCTTCCTAAAGTGATCACCAGCTGGTAGCCAATCAACGCTTTAGTGATTATGCTAATGATAAATTAATCATGAAattaagaaaataataaaaagaaaaaccaACAATCATTTAAGTGTGGTGTGTTTGCTCATGACTATTTGGCTTATGTAAACTAATCTTAAACAATGTGATGTGTTTGCTGAAAAATTACTATGTGATGCATGTATAAAACAGGATTTATTAATTGTATGTTGGTGTGTGACGTTTtataa

>*HaMADS25*

ATTATTTCTTACGTATACAGTTTGTTTCACAGGAgaaaatctagagagagaaagctagagagagagagagatggggagGGGGAGGATAGAGATAAAGAAAATAGAAAACAATACAAACAGGCAGGTGACATACTCAAAGAGAAGAAATGGGATATTCAAGAAGGCTCATGAGCTCACTGTTCTTTGTGATGCCAAGGTTTCTCTTATTATGTTCTCCAACACAGGAAAGTTCCATGAGTACATCAGCCCTTCTACAACGACTAAGAAGATTTATGATATGTATCAGACTACTCTAGGGTTTGATCTCTGGAGCTCCCATTATGAGAGGATGCAAGAAACATTGAAGAAGCTTAAAGATAGCAACAATAAACTTAGGAGAGAGATCAGGCAAAGAGTGCTTGGTGAAGATTTAGATGGTTTGGACATGAATGATCTAACCATACTCGAGCAACACATGCAAGATTCACTCACGGTTGTGCGAGAACGCAAGTATCACGTGATCAAAACTCAAACCGACACCTGCAGGAAGAGGGTGAAGAATTTGGAGCAAAGAAATTGTAATCTTCGACTAGACTATGACACAATACACCAGTTGGAGAAGAAATATGGCATGGTAGAGAATGAAGTAGGCTACGAGTCCACGATTGCATATTCAAATCTCTACGGCTTTTGTGAGAACCCGAACAACAACATTATCCATGGTTCAGGTTACGAGCCTCATGGTCTTCGCCTCGATTGAGTTTTTAATTGGCGCGATGTAATATACACTGGCTATTGGTGGTTAAAGTGGTCATTAGAAGTATGTTAACTAAGTAGCTAGCTAGTTAGCTGATCAGTGCTGACCATAATGTGACAAAGTTTATCTATAACTCTTTATTTATGGATATTGAGAAAACTTT

>*HaMADS26*

AGACATAACTCTTTTGGCATCTAGGTTAATATTATTGTGTATGTATCATGTTTATATAACAAGTTCATCCCCTTTTTGCTGAAACATAGAAGCAATTGGTGAAAGAAGCAACACCCAACCCACCCATACATATCATAAATCATTCACTTCTAGAAAGAAACAACAACAGATTcacaactttccttttcttgccAAGAGTATGGGTAGAGGGAAGGTACAACTAAGGAGGATAGAGAACAAGATAAACAGGCAGGTAACTTTCTCAAAGAGGAGAGGTGGCTTATTGAAGAAAGCCCATGAGATTTCAGTTCTTTGTGATGCTGAAGTTGCCTTAATTGTCTTCTCCAACAAAGGAAAACTATTTGAGTTTTCTACTGACTCTTGCATGGAGAGCATCTTGGAGCGATATGAGAGATATTGTTACTCCGAAAGACAACTAGTCGCAACAGATGCTACTCCGAGAAGCTGGACCCTAGAATACAACAAACTTAAATCTAGGGCTGAGCTATTACAAAGAAATCACAGGCACTATATGGGTGAAGATATCGAGTCACTGAGCTTGAAAGAAATCCAAAATTTGGAGCAACAGCTTGATACTGGACTTAAGAATATTCGTGCAAGAAAAGTACGAAAGCATGCGCGCCTAGAGTATTTAATTAATTTGTTACAAATAGTAGTTTTAAATCATAGATTAACTAGAAGTTTTGTCTTGCAGAACCAACTCCTGCATGAATCAATCAATGAGCTCCAGAAAAAGGGAAAGGCCATACAGGAGCAGAACACTACTTTGACAAAGCAACTCAAGGAGAAGGAGAAAGACAAGACCATACCGCAAAACACTCAATGGGAACAACATAACTATGTGGACCATGATACAACCTTCTTCTTGCCACAGCCACATCCCGCTCTCAACATCGGAGGAGATTACAACCAAGCTACTACTTCTGCTGCTAGTGGTGGTGAAGGAGCTGATGGAAGGACCAGCCAGCTGGACCTCAGTCTGCAGCCAATATATTCGCACCACCTAAGGTGCTTTCCGTCGTAAAATGATCTCATGAGTCGTGACCTGCCAATGCATTAATGATTATGAATAATGGTTTTGTTAATGACATATTAACGAAGAAAGAACAATATTGAACCATCAGCAATCAAGTGATCTATGTACGATAAAATGGGATCAACATAAACACGTTGGATGTTGATTTATTATGTGCTGTGTGACTATTTATGGCTATGTCTTTTGTTACTGAACCTAATGCATATTGGGTTTGGCTAATA

>*HaMADS27*

TTGAAGAGCCAAGTTTTGTGGGTTataaaagaaaagagaaaagaaaagaagagaagataAAAGAAAGAGATATGGGAAAAGGAAggttagagttgaagagaattgAGAACAAGATAAACAGGCAGGTTACCTTTGCCAAGAGAAGAAATGGTCTTCTCAAGAAAGCTTATGAACTTTCTGTCCTTTGTGATGCTGAAGTTGctctcatcatcttctccactcgTGGCAAACTCTATGAGTTCTCTAGCACCTCTAGCATGCTGAAAACACTTGAGAGGTATGAGAAGTGTAGTTTTGGGCCACCAGAACAAAGAAAGCCAGCCAAGGAGGATCTTCAGGAACAAAGCAGTTACCAGGAATACATGAGGCTTAAAGAACGCTACGATGCTTTGAAACAACTCGAAAGGAATTACTATGGTGAGGAGATTGACTCGTTGACTACCAACGAGCTCGAGTCGCTTGAAAGGCAGCTTCATTGTTCATTGAAACAGATTAGGACAATAAGGACTCAGTCGCTGATCGATAAGCTTTACGAACAACAAAAGATGGAACACCAACTTTACGAGTACAACAAGACGCTTCGACTCAGATTAGATGAAGAAAGCCAAGCAGAGGCACTCCAATGGGATGTACACGCTCACGCACACGCGAATGGAATGGTTTATGGGCATCATCATCAGCACCAAGTGTCTCATCCTGCACATGGTGCGTTTTATCATCCGACTGGCTGCGAAACCACATTGCAAATCGGGTACCAGACTGAACAAATCTCGGGAGCGACCTCGTCGAGCATgagccatcatcatcatcaaatgcaGGGTTGGCCTGCATGAAACAAGATTCTATGTTTGCTTGTTAAATCACCAGACTTAATTAATATTGTGTTGTTGTGGGACATTTGGTCTTAGCTAATTTTTAGTTAatgtgtttatgtttgttgtGACTTTTATCAATCTCTCTCTATGATGTCCCATTGCATTTGAA

>*HaMADS28*

ATATATGGTTAATGTTTTTACATTTGAACAAATAAAGCAAAAAAATTTGATTACATTTGTTACTCATCTCACAATGATGCCAAAAAAGAGTAAAGGTCGCCAAAAGATCCAAATGGCAAGAATGGAGAAAGAAAGCAACTTATTGGTAACCTTCTCCAAACGTCGTTCGGGTCTGTTCAAGAAAGCGAGTGAGCTTTGCATCTTGTGCGGGGTGGAAATCGCAATCATTGTTTTCTCTCCGGGGAAAAAGGTATTCTCCTTCGGTCATCCATCTGTAGAGATTATCGTTGATCGTTTTCTCAAGCAAAATCCACCACTGTATTCAAGCACGACGCAACTTATGGAGGCACATCGAAATGCCAATATCCATGAACTCAATAGGCAACTCGCCTATGAGATTAGTCATTTGGAGATTGAGAAGAACAAAAGTGAAGAGCTTACAAAGATTAGGAAAGAAGGTATTGACAATCATTGGTGGGAAGCACCAATAGAGAGCCTAGGCATAGAAGAGCTTGAGCGGTTGAAGGTGGCTATGGGGTTTCTAAAAAAAGACATTGAGGAACAGAAGAAGAGGATCGTGGCGGCTAACCCACTCCAAATCGTTCCAGTGTCTGCTCCCAATGGTATCGGTGGCGAGTACATTGCTAAGGGTTCTGGACTTGATTTAGCAATGACTCCACATGGTTTTGTGCTTGGATTTGGAAATTTCAGTCGCTGAGTCAGATGACGAACAGGGTGGAGACGGTGATCCATCCGAGTAATTTGAGAGTGTGTTAAGTTCATGAAGTTGTTGATGCTATTGCAAAGGAGTAAATTTTCCATCAAATTTCAAAGTTATTCCTGTACTGTTATTTAAAGTATTCTATGAAACATTACTTGTTACCCCCTAAATTTAATAACTAGAATTGAGTTG

>*HaMADS29*

GCTCAATGGTTATGTACCAGTTCATTGAGGATGAACGCGTCAGGAATGTAACCATGAGGAAGAGGAAGGCGAGTCtaatgaagaagatgaatgaGTTGAAGATTTTGTGTGATGTTGATGCATGTCTTGTGATGTATGAAACTGACGACAGTCCACCTGATGTTTGGCCATCTCCTTCTGAAGCGCATCGCGTGATTCAAAAGTTTAATAAATCGCCAATGTGGAGTTCTAATGCCTTACAAGATCACTCAGCTTATCTTCAAAAACAAATAGCCAAAATGAAGAAACAGTTGGCAAAGGAAAAGGAGAAGAACATGAAACATTTAATGGTGAAATGTTTGTTTGATGAGAACGCACTCAGGGAGGTCAACAATCAAGAGGTTTTGGATGGTGTGCGTTCTGCGATTGACAGTGAGATTGAAGCAATTGATGACATGATTAAGGAAGCTCAAGAGAAGGCCAAGAAAAAGATGGTTTTTAGAACCTTCTTTTAAGAATATGTGATCTGTATGCGATCAAATCGCTATGTT
