# Supplementary material for: HaMADS3, HaMADS7, and HaMADS8 are involved in petal prolongation and floret symmetry establishment in sunflower (Helianthus annuus L.)
Source: PeerJ. 2024 Jul 2;12:e17586. doi: 10.7717/peerj.17586 (PMC11225715; doi:10.7717/peerj.17586)
Supplement: Supplemental Information 12 [file peerj-12-17586-s012.docx]

**Supplementary Table 9 The** **MIQE checklist for qPCR**

| Item | | Details |
| --- | --- | --- |
| Experimental design | 1. Definition of experimental and control groups. | The tissues of *lpm* were used as experimental groups, while that of WT were used as control groups. |
|  | 2. Number within each group. | All materials used in this study for qPCR were gathered from 15 individual plants, which were grown in the same conditions, and then mixed for RNA extraction. |
| Sample | 1. Description. | The disc florets of WT and *lpm* at different stages were collected for exploring the temporal expression pattern of MADS-box genes. The petals, pistils, and bracts in disc floret of WT and in ray-like floret of *lpm* were used to investigate the spatial expression patterns of these genes, the petals from ray and disc florets in WT were applied to explore the function of candidate MADS-box genes during the process of petal development, and the petals, in the 1^st^, 5^th^, 15^th^, and 19^th^ parastichy of floret in WT and *lpm* plants, were collected for analyzing the expression level of MADS-box genes in petal on different positions (See details in Table S6). |
|  | 2. Volume/mass of sample processed. | All materials were harvested at least 0.5 g. |
|  | 3. If frozen, how and how quickly? | All materials were immediately frozen in liquid nitrogen. |
|  | 4. If fixed, with what and how quickly? | Not fixed. |
|  | 5. Sample storage conditions and duration (especially for FFPEb samples). | All samples were stored at -80 °C for no more than three months. |
| Nucleic acid extraction | 1. Procedure and/or instrumentation. | Total RNA was extracted by E.Z.N.A®Plant RNA Kit (R6827, Omega, Norcross, GA, USA). |
|  | 2. Name of kit and details of any modifications. | Total RNA was extracted by E.Z.N.A®Plant RNA Kit (R6827, Omega, Norcross, GA, USA). |
|  | 3. Details of DNase or RNase treatment. | The cDNA was obtained using the PrimeScript™ RT reagent Kit with gDNA Eraser (RR047A, TaKaRa, Shiga, Japan). Reaction for removing gDNA was performed using 10 μL mix including: 2.0 μL 5×gDNA eraser buffer, 1.0 μL gDNA eraser, 1 μg total RNA, and adding RNase free dH_2_O up to 10 μL. Then the 10 μL mix was incubated at 42 ℃ for 10 min. |
|  | 4. Contamination assessment (DNA or RNA). | DNA contamination was assessed by a PCR using total RNA, RNA treated by DNA Eraser, and cDNA as template, and then analyzed via 1% agarose gel electrophoresis. Bright and clear band could be observed only using cDNA as template (Figure S1). |
|  | 5. Nucleic acid quantification. | Table S7. |
|  | 6. Instrument and method. | Total RNA was extracted by E.Z.N.A®Plant RNA Kit (R6827, Omega, Norcross, GA, USA). NanoDrop2000 (ThermoFisher, New York, NY, USA) was used for detecting quantification, OD260/280, OD260/230 and contamination assessment of RNA. 1% agarose gel electrophoresis was used for analyzing RNA integrity via electrophoresis apparatus (Bio-Rad, Hercules, CA, USA) and gel imaging system (Bio-Rad, Hercules, CA, USA), using Tris-Borate-EDTA (BL540A, Biosharp, Hefei, Anhui, China) as buffer and SuperRed (BS354A, Biosharp, Hefei, Anhui, China) as dye. |
|  | 7. Purity (A260/A280). | Table S7 |
|  | 8. RNA integrity: method/instrument. | 1% agarose gel electrophoresis was used for analyzing RNA integrity via electrophoresis apparatus (Bio-Rad, Hercules, CA, USA) and gel imaging system (Bio-Rad, Hercules, CA, USA), using Tris-Borate-EDTA (BL540A, Biosharp, Hefei, Anhui, China) as buffer and Gel Red (BS354A, Biosharp, Hefei, Anhui, China) as dye. |
| Reverse transcription | 1. Complete reaction conditions. | The cDNA was obtained using the PrimeScript™ RT reagent Kit with gDNA Eraser (RR047A, TaKaRa, Shiga, Japan). Reaction for removing gDNA was performed using 10 μL mix including: 2.0 μL 5×gDNA eraser buffer, 1.0 μL gDNA eraser, 1 μg total RNA, and adding RNase free dH_2_O up to 10 μL. Then the 10 μL mix was incubated at 42 ℃ for 10 min. Afterword, RNA reverse transcription was performed using 20 μL mix including: 10 μL mix after removing gDNA reaction, 1.0 μL PrimeScript RT enzyme mix I, 1.0 μL RT primer mix, 4.0 μL 5×PrimeScript buffer 2 and 4.0 μL RNase free dH_2_O. The 20 μL mix was incubated at 37 ℃ for 15 min and 85 ℃ for 5 s. Finally, the 20 μL cDNA solution was diluted 10 times and stored at -20 ℃. |
|  | 2. Amount of RNA and reaction volume. | 1 μg total RNA. The reaction volume for removing gDNA was 10 μL, while RNA reverse transcription was performed using 20 μL mix. |
|  | 3. Priming oligonucleotide (if using GSP) and concentration. | RT primer mix, which was obtained TAKARA from HOLDINGS INC., was used for reverse transcription. See details in TAKARA websites (https://www.takarabiomed.com.cn/). |
|  | 4. Reverse transcriptase and concentration. | PrimeScript RT enzyme mix I, which was obtained TAKARA from HOLDINGS INC., was used for reverse transcription. See details in TAKARA websites (https://www.takarabiomed.com.cn/). |
|  | 5. Temperature and time. | The 20 μL mix was incubated at 37 ℃ for 15 min and 85 ℃ for 5 s. |
|  | 6. Manufacturer of reagents and catalogue numbers. | The cDNA was obtained using the PrimeScript™ RT reagent Kit with gDNA Eraser (RR047A, TaKaRa, Shiga, Japan). |
|  | 7. Storage conditions of cDNA. | The cDNA was stored at -20 ℃. |
| qPCR target information | 1. Gene symbol. | Table S8 |
|  | 2. Sequence accession number. | Table S8 |
|  | 3. Location of amplicon. | Table S8 |
|  | 4. Amplicon length. | Table S8 |
|  | 5. What splice variants are targeted? | Table S8 |
| qPCR oligonucleotides | 1. Primer sequences. | Table S8 |
| qPCR protocol | 1. Complete reaction conditions. | qPCR reaction was performed using 20 μL mix including: 10 μL TB Green Premix Ex Taq II (Tli RNaseH Plus), 1.0 μL forward primer (10 μM), 1.0 μL reverse primer (10 μM), 7.0 μL RNase free dH_2_O, and 1.0 μL cDNA. |
|  | 2. Reaction volume and amount of cDNA/DNA. | 1.0 μL (5 ng) cDNA |
|  | 3. Primer, (probe), Mg2, and dNTP concentrations. | Primer concentrations: forward primer (0.5 μM), reverse primer (0.5 μM). TB Green Premix Ex Taq II (Tli RNaseH Plus), which was obtained TAKARA from HOLDINGS INC., was used for qPCR. See details in TAKARA websites (https://www.takarabiomed. com.cn/). |
|  | 4. Buffer/kit identity and manufacturer. | Quantitative real-time PCR (qPCR) was performed by TB Green^®^ Premix Ex Taq^TM^ Ⅱ (RR820A, TaKaRa, Shiga, Japan). |
|  | 5. Complete thermocycling parameters. | qPCR was performed with following programme: 30 s at 95 ℃, then 40 cycles of 5 s at 95 ℃ and 30 s at 56 ℃. |
|  | 6. Manufacturer of qPCR instrument. | CFX96^TM^ Real-Time System (Bio-Rad, Hercules, CA, USA). |
| Data analysis | 1. qPCR analysis program (source, version). | Bio-Rad CFX maestro 1.1 software (version 4.1.2433.1219, https://www.bio-rad.com/). |
|  | 2. Justification of number and choice of reference genes. | A single reference gene. Before qPCR, its expression stability was analyzed, see details in Review-only info Supplemental Files. |
|  | 3. Description of normalization method. | 2^-∆∆Ct^ method. |
|  | 4. Number and stage (reverse transcription or qPCR) of technical replicates. | 3 technical replicates (qPCR). |
|  | 5. Statistical methods for results significance. | Statistical analysis was performed by one-way ANOVA and Tukey multiple comparison using SPSS 22.0 (https://spss.en.softonic.com/). |
|  | 6. Software (source, version). | SPSS 22.0. |
